# More than just a mental stressor: psychological value of social distancing in COVID-19 mitigation through increased risk perception—a preliminary study in China

**DOI:** 10.1057/s41599-021-00774-1

**Published:** 2021-04-15

**Authors:** Yuanchao Gong, Linxiu Zhang, Yan Sun

**Affiliations:** 1 Key Laboratory of Behavioral Science, Institute of Psychology, Chinese Academy of Sciences, Beijing, China; 2 Department of Psychology, University of Chinese Academy of Sciences, Beijing, China; 3 Laboratory of Ecosystem Network Observation and Simulation, Institute of Geosciences and Resources, Chinese Academy of Sciences, Beijing, China; 4 The United Nations Environment Programme—International Ecosystem Management Partnership, Beijing, China

**Keywords:** Social policy, Psychology

## Abstract

Social distancing is an effective measure to prevent epidemic infections during a pandemic outbreak, but its psychological value in COVID-19 pandemic mitigation remained less detected. Our study fills this gap by conducting a nationwide survey in China between 12 and 25 February (2020), and a follow-up survey targeting the same participants between 25 and 28 March (2020). We have discovered that perceived increased time staying at home, a subjective agency for social distancing, positively predicts not only risk perception of COVID-19 epidemic at the outbreak and eased stage, but also predicts subjective controllability of COVID-19 epidemic at the eased stage. Given that risk perception indicates potential active engagement of preventative behavior and that subjective controllability associating with self-efficacy could promote individual health behavior, this study preliminarily justifies the value of social distancing from the angle of perceptual factors, adding to existing mounting evidence of its effect on physically controlling pandemic spread.

## Introduction

The COVID-19 pandemic is still rapidly raging worldwide. Although scientists around the world are continually working on developing a safe and effective COVID-19 vaccine, “with the mutant highly transmissible strain, it will require 90% of the population to be vaccinated to reach herd immunity”, quoting Dr. Manoj Jain, an infectious disease physician in Memphis.^1^ Before we reach this point current pandemic control still needs parallel strategies such as social distancing, hygiene and contact tracing (Ruppel et al., 2021). Among the variant control strategies, social distancing has provided the backbone of controlling the rampant spread of the coronavirus during the severest times of global outbreak (Jawaid, 2020; Kupferschmidt, 2020) and it should be intermittently applied as an indispensable strategy whenever sporadic regional or seasonal resurgence happens (Kissler et al., 2020). Indeed, social distancing has been proven to be an effective measure to prevent epidemic infections during the pandemic outbreak (Kelso et al., 2009), and a model investigation shows that China’s travel restriction policies delayed the spread of COVID-19 from Wuhan to other cities and reduced case incidence in the first 50 days of the epidemic (Cyranoski, 2020; Tian et al., 2020).

Besides its effect on physically controlling the virus spread, social distancing has raised general awareness of and attention to its negative psychological impacts, such as anxiety, depression, loneliness or other mental health problems (Brooks et al., 2020; Miller, 2020). However, it does not necessarily indicate that social distancing bears no positive psychological outcomes. In the following sections, we refer to two hypothesized individual perceptual factors that are highly likely to be brought about by social distancing and that might greatly benefit COVID-19 outbreak control—pandemic risk perception and controllability perception.

Generally, risk perception refers to an individual’s subjective judgment of the characteristics and severity of a risk; adapting to the context of COVID-19 outbreak, given the significance of its multidimensional social, environmental, health and economic impacts (Gautam and Hens, 2020), we define pandemic risk perception as people’s perceived severity and the level of overall impact it would cause to human society. According to social-cognitive models of health behavior change, risk perception plays an indispensable role in health behavior (Schwarzer and Ralf, 2010); specifically, extensive research has pointed out that increased public risk perception of the pandemic is associated with preventative health behavior (Brewer et al., 2007; Motta Zanin et al., 2020; Wise et al., 2020).

Controllability perception, or perceptions of control, consists of multiple dimensions including individual beliefs about the effectiveness of recommended responses in reducing risks, and personal abilities to perform these responses (Norris et al., 1999). According to these, we define the pandemic controllability perception as personal beliefs about the validity of required pandemic controlling strategies such as maintaining interpersonal distance or wearing masks and beliefs that they could make an individual contribution to carry out these behaviors. Closely related to self-efficacy (Krueger and Dickson, 1993), controllability perception is essential for the performance of actual preventive actions against infectious diseases (Sobkow et al., 2020). That is, if a situation is perceived as controllable, it is then recognized as an opportunity, thereby, promoting action (Schwarzer and Ralf, 2010).

Both risk perception and controllability perception can be positively affected by personal experience of risky events. For one thing, past experience would serve as a cognitive source of information and as an affective impression to shape current appraisal of risk situation (Demuth et al., 2016). In addition, Bandura’s self-efficacy mechanism (SEM) claims that controllability is positively associated with individual self-efficacy perception, which could be enhanced by mastery or a successful personal experience (Bandura, 1977; Bandura, 1982). In the study of public responses to hurricane risks, risk perception and perception of control have been proven to be important mediators linking personal past hurricane experience and behavioral intentions to confront the hazard (Demuth et al., 2016). Extending this to the current COVID-19 pandemic, we presume that social distancing would serve as a unique experience influencing risk perception and controllability perception of the pandemic.

Unlike other pandemic control strategies, such as vaccine development, which involves mostly the researchers, or routine tracking that is basically automatic and do not require contribution from an individual, social distancing is a large-scale measure that involves every member of the society, making it a salient experience of living amid the pandemic. Meanwhile, it would be impossible and unacceptable for everyone to acquire direct experience of the severity of the pandemic through infection, thus making the indirect experience a salient alternative to perceiving the severity of the pandemic. Taking climate change as an analogy, given its slow progress, people hardly can have a clear and direct impression of climate change, except for a personal experience of local temperature change (Howe et al., 2013) or negative consequences such as floods (Spence et al., 2011), which contributes to increased beliefs of and concern for climate change and willingness to mitigate climate change. Similarly, regarding social distancing as a consequence of the pandemic to some extent, we postulate that it would enhance public risk perception and the longer people are required to stay at home, the higher the risk they would perceive towards the severity of the pandemic.

We postulate that social distancing would also benefit individual’s controllability perception based on the following three reasons. First, SARS-CoV-2 spreads through the respiratory droplets that would require certain proximity of people, and interpersonal social distancing will effectively reduce the chance of transmission (Wilder-Smith and Freedman, 2020), creating a more favorable environment and buying more time for the control of the pandemic. Second, individuals usually lack beliefs that a single person can control the spread of the pandemic alone while they have firm faith in the government, believing that the government could take the pandemic under control through strict restrictions (Lohiniva et al., 2020). In this sense, social distancing should be taken as a trustworthy authoritative strategy of pandemic control, consistent with our definition of controllability concerning individual trust in the effectiveness of the responsive strategy. Furthermore, people could always get in-time feedback of the positive effects of social distancing from social media presuming local lockdown has been carried strictly, thus creating a sense of “mastery” in terms of making a personal contribution to the pandemic control. This would lead to the increased efficacy perception that would further boost individual faith in controllability of the pandemic, in accordance with the other aspect of our definition of controllability regarding the belief of effectiveness of personal responses.

In this study, we intended to examine whether social distancing would have a positive association with risk and controllability perception of COVID-19 epidemic. For this reason, we conducted two online survey studies in China during the COVID-19 outbreak and eased stages to detect both instant and lasting effects of perceived increased time staying at home due to social distancing on risk perception and controllability perception of COVID-19 pandemic.^2^

Our first survey was conducted between 12 and 25 February (2020) (T1, roughly 3 to 4 weeks after social distancing measure was initially implemented when domestic pandemic remained severe) on an online platform Credamo and collected a total of 1,499 valid responses. The survey respondents’ demographical information is presented in Table 1. One month later, 25–28 March (2020) we conducted a second survey—T2. At the time of T2, the majority of cities had lowered emergency level of the pandemic due to an effective local response to the pandemic and started to loosen social distancing policy. At T2 we surveyed the initial 1499 respondents again, and some adjustments to the questionnaire used at T1 was made according to the real-time national COVID-19 pandemic process. 1,266 of the respondents fully completed the survey and were successfully matched with their first responses (a re-interview rate of 84.46%).

**Table 1:** Demographical information of recipients at T1 (*n* = 1,499) and T2 (*n* = 1,266).

	(T1) *n* (%)	(T2) *n* (%)^a^
Sex		
Male	737 (49.2%)	621 (49.1%)
Female	762 (50.8%)	645 (50.9%)
Education		
Junior high-school and below	56 (3.7%)	36 (2.8%)
Senior high-school degree	240 (16.0%)	186 (14.7%)
College degree	1,100 (73.7%)	966 (76.3%)
Graduate and above	103 (6.9%)	78 (6.2%)
Monthly income (CNY)		
Less than 3,000	383 (25.6%)	290 (22.9%)
3,000–6,000	528 (35.2%)	456 (36.0%)
6,000–10,000	455 (30.4%)	409 (32.3%)
10,000–30,000	128 (8.5%)	108 (8.5%)
More than 30,000	5 (0.3%)	3 (0.2%)
Careers		
Student	336 (22.4%)	263 (20.8%)
Employee in companies	660 (44.0%	591 (46.7%)
Employee in institutions	168 (11.2%)	146 (11.5%)
Self-employed household	214 (14.3%)	181 (14.3%)
Farmer	34 (2.3%)	20 (1.6%)
Others	87 (5.8%)	65 (5.1%)
Place of domicile		
Urban	1,127 (75.2%)	972 (76.8%)
Rural	372 (24.8%)	294 (23.2%)
Age (in years)^b^		
≤29	880 (58.7%)	732 (57.8%)
30–39	497 (33.2%)	442 (34.9%)
≥40	118 (7.9%)	89 (7.0%)
Current residential province		
East China	291 (19.4%)	270 (21.3%)
South China	125 (8.3%)	109 (8.6%)
North China	301 (20.1%)	264 (20.9%)
Central China^c^	499 (33.3%)	402 (31.8%)
Northeast China	54 (3.6%)	45 (3.6%)
Southwest China	101 (6.7%)	73 (5.8%)
Northwest China	128 (8.5%)	103 (8.1%)
Current residential city		
Not Wuhan	1,379 (92.0%)	1,173 (92.7%)
Wuhan	120 (8.0%)	93 (7.3%)

^a^The second survey only targeted those who had completed the first batch.

^b^Four recipients did not answer this question at T1, and three of them entered the survey at T2, thus the variable “age” had three missing values at T2.

^c^Central China has the largest proportion of the respondents due to our request to include as many Wuhan respondents as possible.

## Methods

### Survey sampling planning and procedure

We attempted to acquire a random national sample of 1,500 recipients through an online survey platform Credamo where registered and active users from across China were targeted in this survey. Specifically, since Wuhan was the epicenter of COVID-19 epidemic in China, we required the platform to include Wuhan sample as much as possible within its sample pool. The final sample composition is presented in Table 1. The two batches of the survey were published on Credamo between 12 and 25 February (2020) (T1), and between 25 and 28 March (2020) (T2) with the second survey only targeting the respondents who successfully completed the T1 survey. This work received Ethical Approval from the Chinese Academy of Sciences.

### Questionnaire

The survey questionnaire for this study consisted mainly of three parts: demographical information, psychological covariates, and key variables of our major interest. Owing to the real-time process of domestic COVID-19 pandemic, we added and modified some variables in the second batch of the survey.

#### The first survey

The basic demographical variables included sex, age, education, monthly income, career, place of domicile, current residential city and current residential province. As for the psychological covariates, since risk perception could also result from cognitive and affective processes (Slovic et al., 2013), and the major source for the general public to acquire daily updates COVID-19 epidemic relies greatly on media information, we designed three questions to measure attention to epidemic-related information with answer options ranging from 1 to 7 (1 = never, 7 = always). The questions were as follows: 1. Recently, how often do you browse COVID-19 epidemic related information? 2. Recently, how often do you forward COVID-19 epidemic related information in social media? 3. Recently, how often do you talk about COVID-19 epidemic with families and friends? Meanwhile we believed that conscious reflection and rumination on the reason of this pandemic was an important cognitive process along with a daily focus on media reports, so we also included five questions aiming at examining this cognitive process. The questions were as follows: 4. Recently, how often do you think about the relationship between COVID-19 epidemic and wild animal consumption? With answer options from 1 to 7 (1 = never, 7 = always) 5. Recently, how often do you think of how should human beings get along with nature? (1 = never, 7 = always) 6. Recently, how often do you think of how should human beings get along with wild animals? (1 = never, 7 = always) 7. How thorough are you when thinking of Question 4–6? (1 = not at all; 7 very much) 8. How long would it last every time you think of Question 4–6? (1 = very short; 7 = very long). The last item directly asked about knowledge of COVID-19 pandemic: 9. How much do you know about COVID-19 epidemic? (1 = not at all; 7 = very much). Altogether, these nine items were averaged to compose an integrated indicator of cognitive factors (*α*_T1_ = 0.834; *α*_T2_ = 0.863). As for affective covariates, although “the affect heuristic” tends to define affect as the specific quality of “goodness” or “badness” (Skagerlund et al., 2019; Slovic et al., 2007), research has also pointed out distinct roles of specific emotions in risk perception (Yang and Chu, 2018). Therefore, in this study, we measured five discrete negative emotions (fear, anxiety, anger, disgust, sadness) by asking participants “How much XXX do you feel about current COVID-19 epidemic?” (1 = not at all, 7 = very much) (Yang and Chu, 2018). The three major variables of interest were: (1) risk perception, measured with three items (1. How severe do you think COVID-19 epidemic is? (1 = not at all; 7 = very much) 2. How much do you think other people’s perception of severity of COVID-19 epidemic is? (1 = not at all; 7 = very much) 3. How much do you think the impact of COVID-19 epidemic on the society would be? (1 = no impact at all; 7 = very huge impact) (*α*_T1_ = 0.732; *α*_T2_ = 0.774)); (2) perceived controllability, measured with a single item: How controllable do you think COVID-19 epidemic is? (1 = not at all; 7 = very much); and (3) perceived increased time staying at home (PIT) also measured with a single item: Compared with the time before COVID-19 outbreak, how much do you think your time staying at home has increased? (1 = no change; 7 = a lot of increase).

#### The second survey (the follow-up survey)

The psychological covariates and three major variables of interest were identical with those at T1. Additionally, we measured whether there were any imported cases to each recipient’s current residential city, because as COVID-19 evolved into worldwide pandemic, imported cases posed an increasing threat to the mitigation of domestic epidemic control in China.

## Results

All analyses were performed in SPSS 21.0 with a two-tailed alpha = 0.05. Our major variables of interest were two outcomes: risk perception and perceived controllability of COVID-19 epidemic (hereafter these two outcomes would be briefed as “risk perception” and “controllability perception”, respectively) and one predictor, perceived increased time staying at home (PIT). The descriptive statistics and comparisons of major variables of interest between T1 and T2 are presented in Table 2 and other psychological covariates were presented in Table S1 (see Supplementary Information).

**Table 2:** The descriptive statistics and comparisons of major variables of interest between T1 and T2 (paired samples: *n* = 1,266).

	*M* (s.d.) (T1)	*M* (s.d.) (T2)	*t*	d.f.	*P*
Risk perception	6.17 (0.796)	6.08 (0.832)	4.365	1,265	<0.001
Controllability perception	5.45 (1.234)	5.54 (1.080)	−2.630	1,265	0.009
Perceived increased time staying at home (PIT)	5.97 (1.415)	5.85 (1.183)	2.747	1,265	0.006

Table 2 presents the overall changes of recipients in PIT, risk perception and controllability perception from T1 to T2. The predictor PIT (*t* (1,265) = 2.747, *P* = 0.006) and dependent risk perception (*t* (1,265) = 4.365, *P* < 0.001) decreased significantly at T2, but perceived controllability was higher at T2 (*t* (1,265) = −2.630, *P* = 0.009). These indicate that at T2, recipients experienced less risk, higher controllability of COVID-19 pandemic and fewer perceived time being restricted at home, all in accordance with real-time domestic mitigation of the COVID-19 epidemic. Notably, one-sample *t*-test indicated overall high level of risk perception, controllability perception and PIT at both T1 and T2 (scores were all significantly greater than 4, *P*s < 0.001), indicating that COVID-19 epidemic still was having a great psychological impact.

In order to examine the short-term and long-term effects of PIT on risk perception and perceived controllability, we regressed risk perception (T1 & T2, respectively) and perceived controllability (T1 & T2, respectively) on PIT (T1) using Hierarchical Multiple Regression as shown in Table 3 (the full results were presented in Table S3 in Supplementary Information). After controlling for demographical variables and other psychological covariates, at T1, PIT was positively associated with risk perception (*B* = 0.053, 95%CI [0.026, 0.079], *t* = 3.871, *P* < 0.001) but had no significant association with perceived controllability (*P* > 0.05). At T2, PIT was still a significant predictor (T1). Specifically, the longitudinal regression controlled for demographical variables, psychological covariates (T1 & T2) and corresponding dependent variable measured at T1 following Mouchacca et al.’s (2013) methods. That is, in the longitudinal regression analysis with risk perception (T2) as the outcome, risk perception at T1 was additionally included as a covariate. Perceived controllability was analyzed in the same way. The results revealed that PIT at T1 had longitudinal positive associations with both risk perception (*B* = 0.065, 95%CI [0.037, 0.093], *t* = 4.578, *P* < 0.001) and perceived controllability (*B* = 0.045, 95%CI [0.008, 0.083], *t* = 2.363, *P* = 0.018).

**Table 3:** Partial results of cross-sectional and longitudinal Hierarchical Multiple Regression (predictor: PIT).

Outcome	*B*	95% CI	*t*	*P*	Δ*R*^2^
T1 (*n* = 1,499)					
Risk perception	0.053	[0.026, 0.079]	3.871	<0.001	0.008
Perceived controllability	0.034	[−0.009, 0.077]	1.550	0.121	0.001
T2 (*n* = 1,266)					
Risk perception	0.065	[0.037, 0.093]	4.578	<0.001	0.011
Perceived controllability	0.045	[0.008, 0.083]	2.363	0.018	0.003

## Discussion

### Implications of values of social distancing

Our results show that in China, perceived increased time staying at home as a perceptual response to social distancing policy has not only an instant effect but also a lasting positive effect on epidemic risk perception and thereby would probably bear beneficial results in promoting individual self-protective health behavior. Importantly, these results were controlled for important and well-proven risk perception antecedents in terms of cognitive and emotional processes (Slovic et al., 2013; Yang and Chu, 2018), indicating the indispensable contribution of social distancing to the pandemic response. Based on this statistical relationship, we believe that during social distancing when people have increased risk perception, it would be beneficial to offer the general public with authoritative guidance through social media on how to correctly protect selves and families so that rampant spread of rumors and misinformation during COVID-19 outbreak (Tasnim et al., 2020) would not misguide their preventative health behavior or hamper public trust in government policies and public health measures (Depoux et al., 2020).

However, perceived increased time staying at home did not have a significant short-term association but only a positive association with perception of epidemic controllability at the pandemic eased stage, implying the long-term value of social distancing in terms of cultivating public confidence in controlling the pandemic. Like threat perception, perceived self-efficacy is also an indispensable antecedent of preventative behavior according to one of the most prominent frameworks of public health, the Health Belief Model (HBM) (Sheppard and Thomas, 2020). We believe the long-term positive association would be promising for maintaining public belief in the health benefit of conducting self-protective health behavior as the severity of COVID-19 epidemic gradually decreases.

Essentially, our predictor PIT was a perceptual variable instead of measurement of actual time staying at home, and comparison of PIT between people in Wuhan and People not in Wuhan at both T1 and T2 revealed no significant difference (T1: *t*(1,497) = 1.839, *P* = 0.066; T2: *t*(1,186) = 0.849, *P* = 0.396). These results add to the current knowledge that despite executed with much more strictness, social distancing did not increase PIT in Wuhan. This offers insight for pandemic control worldwide, providing potentially more flexibility of policy making and adjustment.

Currently, the majority of research highlights the negative mental health consequences of social distancing (Brooks et al., 2020; Miller, 2020). However, it seems that the negative emotional responses during COVID-19 epidemic could have been at least connected with epidemic risk itself to some extent and not necessarily directly originated from social distancing, as indicated by the cognitive appraisal frameworks indicating that risk or threat perception has a negative relationship with psychological well-being (Li et al., 2020; Peacock and Wong, 1990). Thus, we examined the relationship between PIT and the negative emotions, which was the average score of five items measuring negative emotions (*α*_T1_ = 0.841, *α*_T2_ = 0.896), and after controlling for negative emotions at T1, no significant association was found between PIT at T1 and negative emotions at T2 (*B* = −0.009, 95% CI [−0.053, 0.035], *t* = −0.400, *P* = 0.689). Besides, research has shown that even at the very beginning stage of social distancing policy implementation when the impact of social distancing was yet to manifest (Wuhan lockdown and other cities’ travel restrictions, which implies that), COVID-19 outbreak already yielded several negative emotional responses such as anxiety, depression, indignation (Li et al., 2020). Furthermore, our study also discovered that with the mitigation of COVID-19 pandemic, negative emotions would decline (see Table S1 in Supplementary Information) and that at T2, none of the five negative emotions showed a significant difference between people in Wuhan and people not in Wuhan even though Wuhan was still under strict lockdown. As a result, we recommend that pandemic control through the implementation of social distancing should not be held back simply because of concern for negative psychological responses. Instead, the proper strategy should be to directly target negative emotions and offer mental care and support to those who are in need during social distancing.

### Further considerations

Based on our results, we hope that experience of strict social distancing would have equipped people with readiness to cope with a further threat in China when imported cases took the place of indigenous cases becoming a greater threat to COVID-19 mitigation in the “post-pandemic era”. The results also offer some valuable reference to other countries and regions that are still going through the pandemic resurgence and strengthening social distancing. In addition, there is still no information currently when exactly this pandemic would eventually come to an end, hence the long-term effect of social distancing on risk perception, perceived controllability or other psychological dimensions should address more attention in the ongoing course of COVID-19 pandemic battle.

To sum up, we investigated the psychological impact of social distancing from the angle of important precautionary health behavior antecedents, risk perception and controllability perception, and discovered positive associations, which have attained little attention before. Particularly, we justified social distancing by showing an invariant level of perceived home staying time of people in regions under different policy strictness and by clarifying the irrelevance of PIT and negative emotions. In conclusion, social distancing has been of great value in the mitigation of COVID-19 pandemic, and further research should give more insights.

## Supplementary information

Supplementary Information

## Data Availability

We have uploaded our data used in the study onto OSF (https://osf.io/ub296/?view_only=96950cfce0ac4237b01a75274a55a05e).

## Footnotes

1 https://www.commercialappeal.com/story/opinion/2021/01/14/how-does-our-immune-system-work-fight-covid-q-a-dr-manoj-jain/4151177001/.

2 Only on March 11 (2020) the World Health Organization (WHO) reclassified the international coronavirus (COVID-19) outbreak a “pandemic”. Therefore, at T1 we could not use “pandemic” and, in order to maintain consistency, we focused on risk perception of COVID-19 as epidemic instead of as pandemic.
