# Correction: More than just a mental stressor: psychological value of social distancing in COVID-19 mitigation through increased risk perception—a preliminary study in China

**DOI:** 10.1057/s41599-021-00962-z

**Published:** 2021-11-15

**Authors:** Yuanchao Gong, Linxiu Zhang, Yan Sun

**Affiliations:** 1 Key Laboratory of Behavioral Science, Institute of Psychology, Chinese Academy of Sciences, Beijing, China; 2 Department of Psychology, University of Chinese Academy of Sciences, Beijing, China; 3 Laboratory of Ecosystem Network Observation and Simulation, Institute of Geosciences and Resources, Chinese Academy of Sciences, Beijing, China; 4 The United Nations Environment Programme—International Ecosystem Management Partnership, Beijing, China

**Keywords:** Social policy, Psychology

Correction to: *Humanities and Social Sciences Communications* 10.1057/s41599-021-00774-1, published online 15 April 2021.

The original paper lacked a clear statement on ethical approval and informed consent. The following statements have now been added to the paper to provide additional clarity for the reader:

**Ethical approval**

Approval was obtained from the ethics committee of Institute of Psychology, Chinese Academy of Sciences. The procedures used in this study adhere to the tenets of the Declaration of Helsinki.

**Informed consent**

At the beginning of the questionnaire, we informed each participant of their rights and welfare, and committed to safeguarding their personal information. Only when participants gave permission to be part of this research would the survey proceed.

