# Supplementary material for: More than just a mental stressor: psychological value of social distancing in COVID-19 mitigation through increased risk perception—a preliminary study in China
Source: Humanit Soc Sci Commun. 2021 Apr 15;8(1):98. doi: 10.1057/s41599-021-00774-1 (PMC8593396; doi:10.1057/s41599-021-00774-1)
Supplement: Supplementary file 1 — Supplementary Information [file 41599_2021_774_MOESM1_ESM.docx]

**More than just a mental stressor: Psychological value of social distancing in COVID-19 mitigation through increased risk perception—a preliminary study in China**

**Yuanchao Gong^a,b^, Linxiu Zhang^c,d^, Yan Sun*****^a,b^**

1. Key Laboratory of Behavioral Science, Institute of Psychology, Chinese Academy of Sciences, Beijing 100101, China.
2. Department of Psychology, University of Chinese Academy of Sciences, Beijing, 100049, China
3. Laboratory of ecosystem network observation and simulation, Institute of Geosciences and resources, Chinese Academy of Sciences, Beijing, 100101, China
4. The United Nations Environment Programme – International Ecosystem Management Partnership, Beijing, 100101, China

* Corresponding author: Yan Sun [suny@psych.ac.cn](mailto:suny@psych.ac.cn)

**Table S1** The descriptive statistics and comparisons of psychological covariates between T1 and T2 (paired samples: *n* = 1,266)

|  | *M* (s.d.) (T1) | *M* (s.d.) (T2) | *t* | d.f. | *P* |
| --- | --- | --- | --- | --- | --- |
| **Cognitive covariate** | 5.52(0.803) | 5.36(0.880) | 7.720 | 1,265 | <0.001 |
| **Emotional covariate** |  |  |  |  |  |
| Fear | 4.83(1.440) | 4.45(1.594) | 8.873 | 1,265 | <0.001 |
| Anxiety | 4.68(1.530) | 4.31(1.657) | 8.381 | 1,265 | <0.001 |
| Anger | 4.64(1.693) | 4.17(1.713) | 9.799 | 1,265 | <0.001 |
| Disgust | 4.38(1.646) | 4.03(1.653) | 7.306 | 1,265 | <0.001 |
| Sadness | 5.02(1.528) | 4.48(1.622) | 11.896 | 1,265 | <0.001 |

**Table S2** Comparisons between responses of psychological covariates of people in and not in Wuhan at T1 and T2

|  | T1 (*n* = 1,499) | | | | T2 (*n* = 1,266) | | | |
| --- | --- | --- | --- | --- | --- | --- | --- | --- |
|  | Wuhan  (*n* = 120) | not Wuhan  (*n* = 1,379) |  |  | Wuhan  (*n* = 93) | not Wuhan  (*n* = 1,173) |  |  |
|  | *M* (s.d.) | *M* (s.d.) | *t* (d.f.) | *P* | *M* (s.d.) | *M* (s.d.) | *t* (d.f.) | *P* |
| Cognitive covariate | 5.41(0.990) | 5.52(0.820) | -1.383(1497) | 0.167 | 5.30(0.880) | 5.36(0.880) | -0.698(1,264) | 0.485 |
| Fear | 4.90(1.411) | 4.83(1.463) | 0.491(1,497) | 0.623 | 4.60(1.438) | 4.44(1.606) | 1.022(111) | 0.309 |
| Anxiety | 4.98(1.446) | 4.69(1.549) | 2.061(144) | 0.041 | 4.49(1.411) | 4.30(1.674) | 1.277(114) | 0.204 |
| Anger | 5.20(1.559) | 4.64(1.711) | 3.473(1,497) | 0.001 | 4.26(1.567) | 4.16(1.724) | 0.525(1,264) | 0.600 |
| Disgust | 5.01(1.452) | 4.36(1.671) | 4.654(148) | <0.001 | 4.32(1.490) | 4.01(1.663) | 1.785(1,264) | 0.075 |
| Sadness | 5.32(1.360) | 5.04(1.556) | 1.862(1,497) | 0.063 | 4.57(1.506) | 4.47(1.631) | 0.578(1,264) | 0.563 |

**Table S3**

(a) Cross-sectional Hierarchical Multiple Regression (*n* = 1,499; outcome variable: risk perception)

|  | Step 1 | | | Step 2 | | | Step 3 | | |
| --- | --- | --- | --- | --- | --- | --- | --- | --- | --- |
|  | *B* | 95% CI | *t* | *B* | 95% CI | *t* | *B* | 95% CI | *t* |
| **Sex**  0 = Male  1 = Female | 0.042 | [-0.042, 0.126] | 0.985 | 0.025 | [-0.051, 0.102] | 0.649 | 0.028 | [-0.048, 0.104] | 0.714 |
| **Education^a^** |  |  |  |  |  |  |  |  |  |
| SH | 0.258 | [0.020, 0.497] | 2.122* | 0.229 | [0.013, 0.446] | 2.076* | 0.202 | [-0.014, 0.419] | 1.838 |
| COL | 0.287 | [0.056, 0.517] | 2.434* | 0.297 | [0.087, 0.507] | 2.776** | 0.265 | [0.055, 0.474] | 2.480* |
| AA | 0.421 | [0.138, 0.704] | 2.920** | 0.361 | [0.103, 0.618] | 2.744** | 0.319 | [0.062, 0.577] | 2.435* |
| **Monthly income (CNY)^b^** |  |  |  |  |  |  |  |  |  |
| MI1 | 0.008 | [-0.156, 0.172] | 0.094 | -0.021 | [-0.169, 0.128] | -0.273 | -0.030 | [-0.178, 0.118] | -0.401 |
| MI2 | 0.047 | [-0.131, 0.226] | 0.520 | -0.008 | [-0.171, 0.155] | -0.097 | -0.026 | [-0.188, 0.136] | -0.311 |
| MI3 | 0.015 | [-0.205, 0.235] | 0.136 | -0.070 | [-0.271, 0.131] | -0.685 | -0.089 | [-0.289, 0.111] | -0.872 |
| MI4 | -0.148 | [-0.891, 0.596] | -0.389 | -0.450 | [-1,126, 0.226] | -1.306 | -0.450 | [-1.123, 0.222] | -1.313 |
| **Career^c^** |  |  |  |  |  |  |  |  |  |
| EC | 0.179 | [-0.002, 0.360] | 1.939 | 0.128 | [-0.037, 0.293] | 1.522 | 0.138 | [-0.026, 0.303] | 1.654 |
| EI | 0.137 | [-0.078, 0.352] | 1.250 | 0.002 | [-0.194, 0.198] | 0.019 | 0.015 | [-0.180, 0.211] | 0.154 |
| SHE | 0.164 | [-0.039 0.366] | 1.586 | 0.090 | [-0.095, 0.274] | 0.952 | 0.108 | [-0.076, 0.292] | 1.156 |
| FAR | 0.218 | [-0.101, 0.536] | 1.341 | 0.244 | [-0.045, 0.533] | 1.654 | 0.234 | [-0.054, 0.522] | 1.592 |
| OT | 0.134 | [-0.089, 0.357] | 1.181 | 0.111 | [-0.092, 0.314] | 1.074 | 0.138 | [-0.064, 0.340] | 1.339 |
| **Place of domicile**  0 = Urban  1 = Rural | -0.037 | [-0.139 0.064] | -0.720 | -0.062 | [-0.154, 0.031] | -1.311 | -0.055 | [-0.147, 0.037] | -1.175 |
| **Age** | 0.006 | [-0.001, 0.012] | 1.581 | 0.005 | [-0.001, 0.011] | 1.645 | 0.005 | [-0.001, 0.011] | 1.676 |
| **Current residential city**  0 **=** Not Wuhan  1 **=** Wuhan | 0.045 | [-0.107, 0.198] | 0.584 | 0.010 | [-0.129, 0.149] | 0.140 | -0.002 | [-0.140, 0.137] | -0.022 |
| **Cognitive covariate** |  |  |  | 0.206 | [0.158, 0.254] | 8.477*** | 0.196 | [0.048, 0.243] | 8.025*** |
| **Emotional covariate** |  |  |  |  |  |  |  |  |  |
| Fear |  |  |  | 0.092 | [0.053, 0.130] | 4.651*** | 0.089 | [0.050, 0.127] | 4.519*** |
| Anxiety |  |  |  | 0.031 | [-0.007, 0.068] | 1.609 | 0.028 | [-0.010, 0.065] | 1.456 |
| Anger |  |  |  | -0.006 | [-0.036, 0.024] | -0.378 | -0.002 | [-0.032, 0.028] | -0.122 |
| Disgust |  |  |  | 0.036 | [0.007, 0.065] | 2.477* | 0.034 | [0.005, 0.063] | 2.325* |
| Sadness |  |  |  | 0.060 | [0.028, 0.091] | 3.754*** | 0.055 | [0.024, 0.086] | 3.480*** |
| **PIT** |  |  |  |  |  |  | 0.053 | [0.026, 0.079] | 3.871*** |
| (Constant) | 5.579 | [5.284, 5.875] | 37.068*** | 3.531 | [3.163, 3.899] | 18.804*** | 3.344 | [2.966, 3.723] | 17.325*** |
| *Δ*$R^{2}$ | 0.026 | | | 0.177 | | | 0.008 | | |
| *F* (d.f.1 d.f.2) | 2.511 (16 1,478) | | | 17.081 (22 1,472) | | | 17.149 (23 1,471) | | |
| *P* | 0.001 | | | <0.001 | | | <0.001 | | |

^a^ “Junior high school and below” was set as reference. SH = Senior high school degree; COL = College degree; AA = Graduate and above.

^b^ “Less than 3,000” was set as reference. MI1 = 3,000-6,000; MI2 = 6,000-10,000; MI3 = 10,000-30,000; MI4 = More than 30,000.

^c^ “Student” was set as reference. EC = Employee in companies; EI = Employee in institutions; SHE = Self-employed household; FAR = Farmer; OT = others.

(b) Cross-sectional Hierarchical Multiple Regression (*n* = 1,499; outcome variable: controllability perception)

|  | Step 1 | | | Step 2 | | | Step 3 | | |
| --- | --- | --- | --- | --- | --- | --- | --- | --- | --- |
|  | *B* | 95% CI | *t* | *B* | 95% CI | *t* | *B* | 95% CI | *t* |
| **Sex**  0 = Male  1 = Female | -0.014 | [-0.141, 0.113] | -0.212 | -0.0053 | [-0.126, 0.119] | -0.056 | -0.002 | [-0.124, 0.120] | -0.031 |
| **Education** |  |  |  |  |  |  |  |  |  |
| SH | -0.130 | [-0.491, 0.232] | -0.704 | -0.050 | [-0.397, 0.297] | -0.285 | -0.068 | [-0.415, 0.280] | -0.382 |
| COL | -0.237 | [-0.587, 0.113] | -1.330 | -0.134 | [-0.470, 0.202] | -0.782 | -0.155 | [-0.492, 0.182] | -0.901 |
| AA | -0.516 | [-0.945, -0.087] | -2.360* | -0.351 | [-0.764, 0.062] | -1.688 | -0.377 | [-0.791, 0.036] | -1.789 |
| **Monthly income (CNY)** |  |  |  |  |  |  |  |  |  |
| MI1 | 0.183 | [-0.065, 0.431] | 1.449 | 0.103 | [-0.135, 0.341] | 0.848 | 0.097 | [-0.141, 0.335] | 0.797 |
| MI2 | 0.197 | [-0.074, 0.467] | 1.428 | 0.049 | [-0.212, 0.310] | 0.369 | 0.038 | [-0.223, 0.298] | 0.283 |
| MI3 | 0.423 | [0.090, 0.757] | 2.490* | 0.225 | [-0.097, 0.546] | 1.371 | 0.213 | [-0.109, 0.534] | 1.297 |
| MI4 | 0.577 | [-0.550, 1.704] | 1.005 | 0.430 | [-0.652, 1.513] | 0.780 | 0.430 | [-0.652, 1.512] | 0.780 |
| **Career** |  |  |  |  |  |  |  |  |  |
| EC | -0.138 | [-0.411, 0.136] | -0.985 | -0.157 | [-0.421, 0.107] | -1.170 | -0.151 | [-0.415, 0.113] | -1.120 |
| EI | 0.025 | [-0.301, 0.351] | 0.150 | -0.01 | [-0.333, 0.296] | -0.117 | -0.010 | [-0.324, 0.304] | -0.063 |
| SHE | 0.080 | [-0.226, 0.387] | 0.515 | 0.043 | [-0.253, 0.338] | 0.282 | 0.055 | [-0.241, 0.351] | 0.363 |
| FAR | -0.120 | [-0.602, 0.363] | -0.486 | -0.154 | [-0.617, 0.310] | -650 | -0.160 | [-0.632, 0.303] | -0.678 |
| OT | -0.358 | [-0.695, -0.020] | -2.078* | -0.323 | [-0.648, 0.001] | -1.955 | -0.306 | [-0.631, 0.019] | -1.864 |
| **Place of domicile**  0 = Urban  1 = Rural | 0.010 | [-0.143 0.164] | 0.131 | 0.006 | [-0.153, 0.142] | -0.073 | -0.001 | [-0.149, 0.147] | -0.016 |
| **Age** | 0.018 | [0.008, 0.028] | 3.401*** | 0.015 | [0.005, 025] | 2.938** | 0.015 | [0.005, 0.025] | 2.949** |
| **Current residential city**  0 **=** Not Wuhan  1 **=** Wuhan | -0.517 | [-0.747, -0.286] | -4.393*** | -0.443 | [-0.666, -0.221] | -3.906*** | -0.451 | [-0.673, -0.228] | -3.969*** |
| **Cognitive covariate** |  |  |  | 0.414 | [0.338, 0.491] | 10.639*** | 0.408 | [0.331, 0.484] | 10.401*** |
| **Emotional covariate** |  |  |  |  |  |  |  |  |  |
| Fear |  |  |  | -0.036 | [-0.098, 0.026] | -1.143 | -0.038 | [-0.100, 0.024] | -1.203 |
| Anxiety |  |  |  | -0.074 | [-0.134, -0.014] | -2.411* | -0.076 | [-0.136, -0.016] | -2.474* |
| Anger |  |  |  | -0.056 | [-0.105, -0.007] | -2.264* | -0.053 | [-0.102, -0.005] | -2.157* |
| Disgust |  |  |  | 0.012 | [-.035, 0.058] | 0.486 | 0.010 | [-0.036, 0.057] | 0.434 |
|  |  |  |  |  |  |  |  |  |  |
| Sadness |  |  |  | 0.012 | [-0.038, 0.062] | 0.484 | 0.010 | [-0.040, 0.060] | 0.374 |
| **PIT** |  |  |  |  |  |  | 0.034 | [-0.009, 0.077] | 1.550 |
| (Constant) | 5.123 | [4.676, 5.570] | 22.471*** | 3.600 | [3.011, 4.190] | 11.973*** | 3.480 | [2.871, 4.089] | 11.210*** |
| *Δ*$R^{2}$ | 0.049 | | | 0.081 | | | 0.001 | | |
| *F* (d.f.1 d.f.2) | 4.744 (16 1,478) | | | 10.004 (22 1,472) | | | 9.683 (23 1,471) | | |
| *P* | <0.001 | | | <0.001 | | | <0.001 | | |

(c) Longitudinal Hierarchical Multiple Regression (*n* = 1,266; outcome variable: risk perception at T2)

|  | Step 1 | | | Step 2 | | | Step 3 | | |
| --- | --- | --- | --- | --- | --- | --- | --- | --- | --- |
|  | *B* | 95% CI | *t* | *B* | 95% CI | *t* | *B* | 95% CI | *t* |
| **Sex**  0 = Male  1 = Female | 0.047 | [-0.048, 0.142] | 0.969 | 0.021 | [-0.059, 0.100] | 0.507 | 0.021 | [-0.058, 0.100] | 0.517 |
| **Education** |  |  |  |  |  |  |  |  |  |
| SH | -0.174 | [-0.129, 0.295] | 1.128 | 0.013 | [-0.242, 0.269] | 0.103 | -0.027 | [-0.281, 0.227] | -0.208 |
| COL | 0.043 | [-0.248, 0.179] | 0.291 | -0.099 | [-0.346, 0.148] | -0.789 | -0.145 | [-0.391, 0.101] | -1.157 |
| AA | 0.194 | [-0.155, 0.430] | 1.089 | -0.065 | [-0.361, 0.230] | -0.434 | -0.121 | [-0.415, 0.173] | -0.810 |
| **Monthly income (CNY)** |  |  |  |  |  |  |  |  |  |
| MI1 | 0.022 | [-0.172, 0.216] | 0.223 | 0.054 | [-0.109, 0.217] | 0.65 | 0.039 | [-0.123, 0.201] | 0.474 |
| MI2 | 0.035 | [-0.176, 0.221] | 0.328 | 0.010 | [-0.168, 0.187] | 0.110 | -0.018 | [-0.195, 0.158 | -0.205 |
| MI3 | 0.017 | [-0.239, 0.255] | 0.133 | 0.012 | [-0.205, 0.228] | 0.108 | -0.019 | [-0.234, 0.196] | -0.174 |
| MI4 | -0.031 | [-1.013, 0.815] | -0.062 | 0.175 | [-0.647, 0.998] | 0.418 | 0.182 | [-0.634, 0.998] | 0.437 |
| **Career** |  |  |  |  |  |  |  |  |  |
| EC | 0.095 | [-0.117, 0.336] | 0.879 | -0.038 | [-0.216, 0.140] | -0.421 | -0.019 | [-0.196, 0.158] | -0.208 |
| EI | 0.109 | [-0.139, 0.423] | 0.862 | -0.042 | [-0.251, 0.166] | -0.399 | -0.028 | [-0.234, 0.179] | -0.262 |
| SHE | 0.103 | [-0.134, 0.370] | 0.852 | -0.045 | [-0.244, 0.155] | -0.438 | -0.019 | [-0.217, 0.179] | -0.185 |
| FAR | -0.460 | [-0.870, 0.394] | -2.202* | -0.464 | [-0.808, -0.120] | -2.643** | -0.484 | [-0.826, -0.143] | -2.781** |
| OT | 0.244 | [-0.022, 0.509] | 1.802 | 0.078 | [-0.144, 0.301] | 0.692 | 0.114 | [-0.108, 0.335] | 1.007 |
| **Place of domicile**  0 = Urban  1 = Rural | 0.045 | [-0.072, 0.171] | 0.751 | 0.018 | [-0.080, 0.116] | 0.361 | 0.026 | [-0.071, 0.124] | 0.529 |
| **Age** | 0.004 | [-0.005, 0.010] | 0.856 | 0.004 | [-0.003, 0.011] | 1.186 | 0.004 | [-0.002, 0.011] | 1.261 |
| **Current residential city**  0 **=** Not Wuhan  1 **=** Wuhan | 0.205 | [-0.027, 0.125] | 2.259* | 0.171 | [0.022, 0.321] | 2.248* | 0.158 | [0.009, 0.306] | 2.080* |
| **IMP^a^** | 0.014 | [-0.081, 0.285] | 0.289 | -0.008 | [-0.087, 0.072] | -0.187 | -0.004 | [-0.083, 0.075] | -0.100 |
| **Cognitive covariate (T1)** |  |  |  | -0.076 | [-0.141, -0.011] | -2.282* | -0.091 | [-0.156, -0.026] | -2.736** |
| **Cognitive covariate (T2)** |  |  |  | 0.167 | [0.108, 0.227] | 5.523*** | 0.176 | [0.117, 0.235] | 5.843*** |
| **Emotional covariate**  **(T1)** |  |  |  |  |  |  |  |  |  |
| Fear |  |  |  | 0.029 | [-0.011, 0.070] | 1.413 | 0.029 | [-0.012, 0.069] | 1.393 |
| Anxiety |  |  |  | -0.043 | [-0.083, -0.003] | -2.090* | -0.045 | [-0.085, -0.005] | -2.200* |
| Anger |  |  |  | -0.039 | [-0.071, -0.006] | -2.322** | -0.034 | [-0.066, -0.001] | -2.032* |
| Disgust |  |  |  | 0.003 | [-0.029, 0.034] | 0.163 | 0.001 | [-0.030, 0.032] | 0.054 |
| Sadness |  |  |  | 0.048 | [0.014, 0.082] | 2.745** | 0.041 | [0.007, 0.075] | 2.343* |
| **(T2)** |  |  |  |  |  |  |  |  |  |
| Fear |  |  |  | 0.029 | [-0.012, 0.071] | 1.382 | 0.027 | [-0.014, 0.068] | 1.293 |
| Anxiety |  |  |  | 0.051 | [-0.009, 0.094] | 2.362* | 0.039 | [0.09, 0.093] | 2.362* |
| Anger |  |  |  | -0.026 | [-0.064, 0.011] | -1.384 | -0.027 | [-0.063, 0.011] | -1.398 |
| Disgust |  |  |  | -0.007 | [-0.043, 0.030] | -0.360 | -0.007 | [-0.042, 0.031] | -0.294 |
| Sadness |  |  |  | 0.045 | [0.010, 0.080] | 2.492* | 0.053 | [0.010, 0.080] | 2.515* |
| **Risk perception (T1)** |  |  |  | 0.469 | [0.416, 0.523] | 17.213*** | 0.441 | [0.405, 0.512] | 16.899*** |
| **PIT (T1)** |  |  |  |  |  |  | 0.078 | [0.037, 0.093] | 4.578*** |
| (Constant) | 5.753 | [4.217, 5.123] | 31.462*** | 2.217 | [1.754, 2.680] | 9.399*** | 2.003 | [1.535, 2.471] | 8.394*** |
| *Δ*$R^{2}$ | 0.022 | | | 0.305 | | | 0.011 | | |
| *F* (d.f.1 d.f.2) | 1.652 (17 1,245) | | | 19.978 (30 1,232) | | | 20.322 (31 1,231) | | |
| *P* | 0.046 | | | <0.001 | | | <0.001 | | |

^a^ IMP: “Are there any imported COVID-19 cases in your city?” (0 = No; 1 = Yes)

(d) Longitudinal Hierarchical Multiple Regression (*n* = 1,266; outcome variable: controllability perception at T2)

|  | Step 1 | | | Step 2 | | | Step 3 | | |
| --- | --- | --- | --- | --- | --- | --- | --- | --- | --- |
|  | *B* | 95% CI | *t* | *B* | 95% CI | *t* | *B* | 95% CI | *t* |
| **Sex**  0 = Male  1 = Female | -0.011 | [-0.130, 0.109] | -0.175 | 0.013 | [-0.094, 0.119] | 0.230 | 0.012 | [-0.094, 0.119] | 0.229 |
| **Education** |  |  |  |  |  |  |  |  |  |
| SH | 0.625 | [0.242, 1.099] | 3.202*** | 0.636 | [0.295, 0.977] | 3.657*** | 0.606 | [0.264, 0.947] | 3.480*** |
| COL | 0.310 | [-0.058, 0.679] | 1.653 | 0.400 | [0.071, 0.792] | 2.385* | 0.365 | [0.036, 0.695] | 2.175* |
| AA | 0.277 | [-0.163, 0.717] | 1.235 | 0.502 | [0.109, 0.896] | 2.504* | 0.460 | [0.065, 0.854] | 2.286* |
| **Monthly income (CNY)** |  |  |  |  |  |  |  |  |  |
| MI1 | 0.067 | [-0.178, 0.312] | 0.536 | -0.006 | [-0.224, 0.212] | -0.054 | -0.016 | [-0.233, 0.202] | -0.142 |
| MI2 | 0.212 | [-0.055, 0.478] | 1.559 | 0.072 | [-0.165, 0.310] | 0.599 | 0.053 | [-0.184, 0.291] | 0.440 |
| MI3 | 0.058 | [-0.266, 0.382] | 0.351 | -0.159 | [-0.449, 0.130] | -1.080 | -0.180 | [-0.469, 0.110] | -1.219 |
| MI4 | -0.373 | [-1.613, 0.867] | -0.590 | -0.437 | [-1.536, 0.662] | -0.780 | -0.428 | [-1.524, 0.669] | -0.765 |
| **Career** |  |  |  |  |  |  |  |  |  |
| EC | 0.127 | [-0.140, 0.394] | 0.934 | 0.187 | [-0.051, 0.425] | 1.542 | 0.198 | [-0.039, 0.436] | 1.639 |
| EI | 0.163 | [-0.150, 0.476] | 1.024 | 0.129 | [-0.149, 0.408] | 0.910 | 0.139 | [-0.139, 0.417] | 0.979 |
| SHE | 0.430 | [0.132, 0.729] | 2.827** | 0.376 | [0.110, 0.642] | 2.770** | 0.393 | [0.126, 0.651] | 2.893** |
| FAR | -0.013 | [-0.531, 0.505] | -0.048 | 0.207 | [-0.253, 0.668] | 0.883 | 0.191 | [-0.269, 0.651] | 0.813 |
| OT | 0.099 | [-0.236, 0.435] | 0.582 | 0.121 | [-0.176, 0.418] | 0.800 | 0.143 | [-0.154, 0.440] | 0.945 |
| **place of domicile**  0 = Urban  1 = Rural | 0.017 | [-0.131, 0.165] | 0.227 | 0.016 | [-0.115, 0.148] | 0.246 | 0.023 | [-0.109, 0.154] | 0.337 |
| **Age** | 0.011 | [-0.001, 0.021] | 2.105* | 0.003 | [-0.006, 0.012] | 0.622 | 0.003 | [-0.006, 0.012] | 0.662 |
| **Current residential city**  0 **=** Not Wuhan  1 **=** Wuhan | -0.491 | [-0.716, -0.266] | -4.280*** | -0.320 | [-0.521, -0.119] | -3.125** | -0.331 | [-0.532, -0.130] | -3.234*** |
| **IMP** | 0.013 | [-0.106, 0.133] | 0.218 | 0.043 | [-0.064, 0.149] | 0.785 | 0.045 | [-0.062, 0.151] | 0.827 |
| **Cognitive covariate (T1)** |  |  |  | -0.121 | [-0.209, -0.033] | -2.692** | -0.131 | [-0.220, -0.043] | -2.923** |
| **Cognitive covariate (T2)** |  |  |  | 0.324 | [0.244, 0.403] | 8.006*** | 0.330 | [0.251, 0.410] | 8.160*** |
| **Emotional covariate**  **(T1)** |  |  |  |  |  |  |  |  |  |
| Fear |  |  |  | 0.018 | [-0.036, 0.073] | 0.665 | 0.017 | [-0.037, 0.072] | 0.627 |
| Anxiety |  |  |  | -0.010 | [-0.064, 0.044] | -0.371 | -0.012 | [-0.065, 0.042] | -0.429 |
| Anger |  |  |  | -0.033 | [-0.077, 0.011] | -1.470 | -0.029 | [-0.073, 0.014] | -1.317 |
| Disgust |  |  |  | 0.029 | [-0.013, 0.071] | 1.354 | 0.028 | [-0.015, 0.070] | 1.284 |
| Sadness |  |  |  | 0.027 | [-0.019, 0.072] | -1.151 | 0.021 | [-0.024, 0.067] | 0.911 |
| **(T2)** |  |  |  |  |  |  |  |  |  |
| Fear |  |  |  | -0.008 | [-0.064, 0.047] | -0.299 | -0.010 | [-0.065, 0.045] | -0.357 |
| Anxiety |  |  |  | -0.083 | [-0.140, -0.026] | -2.854** | -0.084 | [-0.141, -0.027] | -2.881** |
| Anger |  |  |  | 0.010 | [-0.040, 0.060] | 0.400 | 0.010 | [-0.040, 0.060] | 0.400 |
| Disgust |  |  |  | 0.002 | [-0.047, 0.052] | 0.095 | 0.004 | [-0.046, 0.053] | 0.143 |
| Sadness |  |  |  | -0.024 | [-0.071, 0.023] | -0.986 | -0.024 | [-0.071, 0.023] | -0.989 |
| **Controllability perception (T1)** |  |  |  | 0.328 | [0.284, 0.373] | 14.439*** | 0.326 | [0.281, 0.370] | 14.333*** |
| **PIT (T1)** |  |  |  |  |  |  | 0.045 | [0.008, 0.083] | 2.363* |
| (Constant) | 4.670 | [4.217, 5.123] | 20.221*** | 2.291 | [1.707, 2.876] | 7.691*** | 2.126 | [1.572, 2.725] | 6.959*** |
| *Δ*$R^{2}$ | 0.076 | | | 0.211 | | | 0.003 | | |
| *F* (d.f.1 d.f.2) | 6.003 (17 1,245) | | | 16.522 (30 1,232) | | | 16.229 (31 1,231) | | |
| *P* | <0.001 | | | <0.001 | | | <0.001 | | |
